# The homeobox transcription factor Even-skipped regulates acquisition of electrical properties in *Drosophila *neurons

**DOI:** 10.1186/1749-8104-1-3

**Published:** 2006-11-16

**Authors:** Edward CG Pym, Tony D Southall, Christopher J Mee, Andrea H Brand, Richard A Baines

**Affiliations:** 1 Neuroscience Group, Department of Biological Sciences, University of Warwick, Coventry CV4 7AL, UK; 2 The Gurdon Institute and Department of Physiology, Development and Neuroscience, University of Cambridge, Tennis Court Road, Cambridge CB2 1QN, UK

## Abstract

**Background:**

While developmental processes such as axon pathfinding and synapse formation have been characterized in detail, comparatively less is known of the intrinsic developmental mechanisms that regulate transcription of ion channel genes in embryonic neurons. Early decisions, including motoneuron axon targeting, are orchestrated by a cohort of transcription factors that act together in a combinatorial manner. These transcription factors include Even-skipped (Eve), islet and Lim3. The perdurance of these factors in late embryonic neurons is, however, indicative that they might also regulate additional aspects of neuron development, including the acquisition of electrical properties.

**Results:**

To test the hypothesis that a combinatorial code transcription factor is also able to influence the acquisition of electrical properties in embryonic neurons we utilized the molecular genetics of *Drosophila *to manipulate the expression of Eve in identified motoneurons. We show that increasing expression of this transcription factor, in two Eve-positive motoneurons (aCC and RP2), is indeed sufficient to affect the electrical properties of these neurons in early first instar larvae. Specifically, we observed a decrease in both the fast K^+ ^conductance (I_Kfast_) and amplitude of quantal cholinergic synaptic input. We used charybdotoxin to pharmacologically separate the individual components of I_Kfast _to show that increased Eve specifically down regulates the Slowpoke (a BK Ca^2+^-gated potassium channel), but not Shal, component of this current. Identification of target genes for Eve, using DNA adenine methyltransferase identification, revealed strong binding sites in *slowpoke *and *nAcRα-96Aa *(a nicotinic acetylcholine receptor subunit). Verification using real-time PCR shows that pan-neuronal expression of *eve *is sufficient to repress transcripts for both *slo *and *nAcRα-96Aa*.

**Conclusion:**

Taken together, our findings demonstrate, for the first time, that Eve is sufficient to regulate both voltage- and ligand-gated currents in motoneurons, extending its known repertoire of action beyond its already characterized role in axon guidance. Our data are also consistent with a common developmental program that utilizes a defined set of transcription factors to determine both morphological and functional neuronal properties.

## Background

The development of electrical properties in embryonic neurons is likely due to factors both intrinsic and extrinsic to the individual cells. Intrinsic factors will be determined by the clonal lineage of individual neurons [1], while exposure to external factors, such as synaptic activity, glia-derived factors and sensory modifications, likely shape the final electrical phenotype [2-5]. Thus, the particular mix of individual conductances expressed by neurons is likely a product not only of regulated gene expression but also of activity-dependent mechanisms that together influence channel function through subcellular distribution, relative density and post-translational modifications [6,7].

A key question that remains to be resolved, however, is how much circuit tuning arises from genetic determination as opposed to activity-dependent mechanisms. Recent modeling studies indicate that extrinsic factors predominate given that indistinguishable network activity can arise from widely disparate combinations of individual conductances [8]. However, these analyses are based on neurons that are already components of functional networks. By comparison, in the early stages of their development, embryonic neurons are largely deprived of extrinsic factors such as synaptic activity and sensory feedback. It is in this relatively early phase of electrical development that genetic determination will likely predominate to ensure, at a minimum, the expression of a default set of ion channel proteins. Only after a neuron has the capability to contribute to a network can other, largely extrinsic factors, begin to shape the final mix of ion channels expressed.

A wealth of studies of motoneuron specification, in a range of animals from worms to mammals, has shown that subclass identity is dictated, in part, by a combinatorial code of transcription factors [9]. For example, in the *Drosophila *embryo, the approximately 36 motoneurons in each abdominal hemi-segment express a stereotypic mix of identified transcription factors that are evolutionarily conserved with mammals [10-14]. Motoneurons that predominantly innervate ventral and lateral muscles express the LIM-homeodomain transcription factor genes *islet *and *lim3 *together with *Hb9*, while motoneurons that project to dorsal muscles express the transcription factor gene *even-skipped *(*eve*) [9,14,15]. Given that motoneuron axon guidance is under combinatorial control of a set of transcription factors, this study addresses whether the acquisition of electrical properties, at least in early development, might be similarly influenced by these same regulatory proteins.

We used targeted gene expression to increase the level of Eve, in just two Eve-positive motoneurons (aCC and RP2), and show that this is sufficient to elicit reductions in both a voltage-gated K^+ ^current and acetylcholine (ACh)-gated synaptic currents. We attribute the reduction in K^+ ^currents to a reduction in the current carried by Slo. We identified binding sites for Eve using DNA adenine methyltransferase identification (DamID). Our analysis identified a discrete set of putative target genes, which includes those encoding for the voltage-gated K^+ ^channel Slo and ligand-gated ACh nicotinic subunit nAcRα-96Aa. That Eve is able to transcriptionally repress these target genes is verified by quantitative PCR (QRT-PCR). Our results show that a transcription factor, known to be required for correct choice of axonal pathfinding, is also sufficient to influence the acquisition of electrical properties in neurons, indicating that both morphological and functional development in embryonic neurons are controlled by common regulatory mechanisms.

## Results

### Increased Eve alters electrical properties in motoneurons

The motoneurons aCC and RP2 express the transcription factor Eve, which is required for appropriate axon targeting during early embryogenesis [15]. This observation forms part of the body of evidence that underpins the hypothesis that a combinatorial code of transcription factor expression orchestrates axon pathfinding and target choice of motoneurons [9,16]. It is notable, however, that *eve *expression can still be detected after these earlier processes have resolved [17], indicating that it may also regulate additional facets of neuron development. One more obvious aspect of late neuronal development is the acquisition of electrical properties.

**Figure 1:**
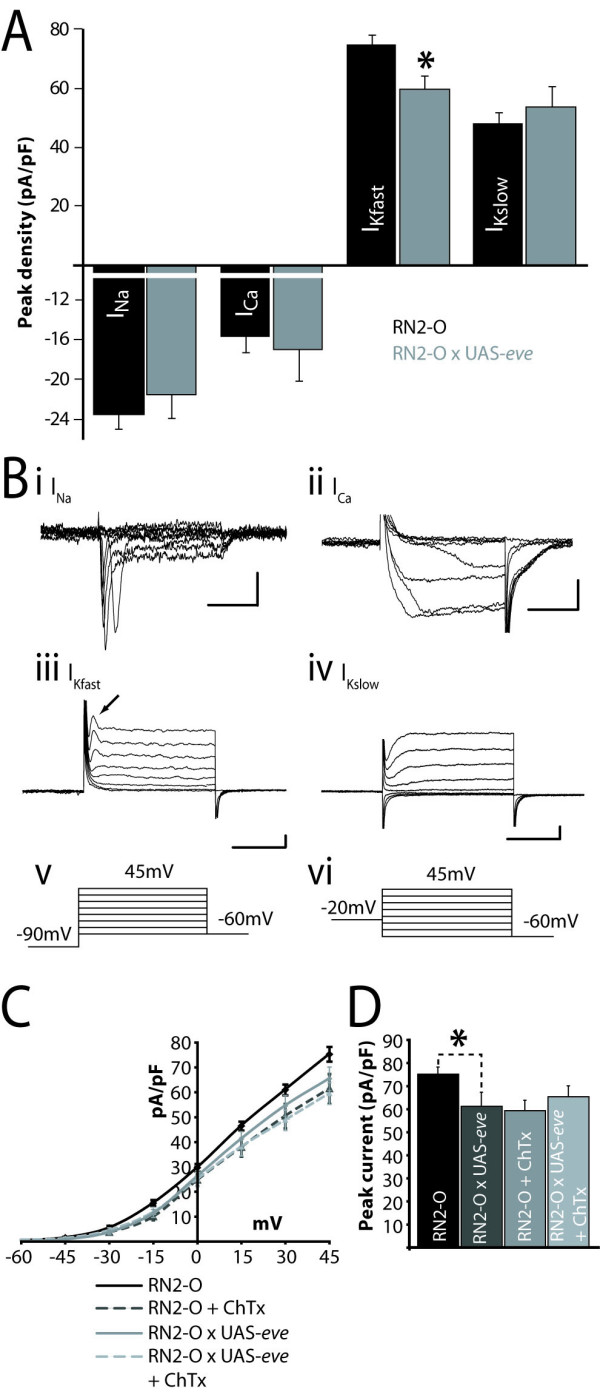
Over-expression of *eve *in aCC/RP2 is sufficient to alter electrical properties. **(A) **Over-expression of *eve *in aCC/RP2 results in a decrease in peak conductance of I_Kfast _(78.6 ± 4.0 versus 62.3 ± 8.4 pA/pF, measured at +45 mV, p ≤ 0.05) and no significant change in I_Na _(-23.4 ± 1.6 versus -21.4 ± 2.2 pA/pF, -15 mV), I_Ca _(-15.7 ± 1.6 versus -17.1 ± 2.9 pA/pF, 0 mV) or I_Kslow _(48.3 ± 3.5 versus 53.7 ± 6.8 pA/pF, +45 mV). **(B) **Example voltage clamp recordings (examples shown from wild-type aCC/RP2) of the four major voltage-gated currents in aCC/RP2; I_Na_, I_Ca_, I_Kfast _(arrow shows current measured) and I_Kslow _(for a more detailed discussion of these conductances see [34]). I_Na_, I_Ca_, I_Kfast _were evoked using the protocol shown (v) while I_Kslow _was evoked using the protocol shown in (vi). **(C) **IV plots for I_Kfast _measured following over-expression of *eve*, with or without ChTx, show no obvious changes in activation kinetics. **(D) **Peak current amplitudes of I_Kfast _(measured at +45 mV) show significant decreases following either over-expression of *eve *or addition of ChTx. Simultaneous over-expression of eve and addition of ChTx does not further decrease I_Kfast_, indicating that expression of *eve *specifically down-regulates the *slowpoke *component of this conductance. For (c,d) n = 8, mean ± SE, *p ≤ 0.05.

To test this hypothesis, we manipulated the expression of *eve *in the motoneurons aCC and RP2 (using RN2-O GAL4) and used whole-cell patch recordings to determine the consequences. Our choice of motoneuron was guided by the fact that aCC/RP2 endogenously express *eve *[18] and manipulation of its expression should, thus, result in altered electrophysiology if this transcription factor contributes to setting electrical properties in these neurons. Over-expression of *eve*, in aCC/RP2, is sufficient to perturb wild-type electrical properties. Of the voltage-gated conductances examined, only the peak fast K^+ ^conductance (I_Kfast_) was significantly reduced (78.6 ± 4.0 versus 62.3 ± 8.4 pA/pF, measured at +45 mV, p ≤ 0.05), while I_Kslow_, I_Na _and I_Ca _were not affected (Figure 1a,b). Analysis of current-voltage (IV) plots for I_Kfast _show no obvious change in activation, as might be expected if the reduction in peak amplitude of this current was due to a change in voltage-dependency of activation (Figure 1c). Control recordings were made from RN2-O/+ (which are identical to the alternative controls of Canton-S wild type and RN2-O GAL4/UAS-GFP; ECG Pym and RA Baines, unpublished data).

Two gene products are known to contribute to I_Kfast _in central neurons in *Drosophila*: Shal and Slo [19,20]. To determine which of these components is affected by over-expression of *eve*, we utilized charybdotoxin (ChTX), which is a selective blocker of Slo in both *Drosophila *neurons and muscle [21]. In the presence of ChTX (200 nM), I_Kfast _in wild-type aCC/RP2 was reduced by 22% (87.9 ± 4.6 versus 61.2 ± 6.0 pA/pF, p ≤ 0.05; Figure 1c,d). Removal of extracellular Ca^2+^, the influx of which is required to activate Slo, resulted in a similar reduction (36%; 55.8 ± 3.2 pA/pF), consistent with ChTX blocking only the Slo-mediated component of I_Kfast _(data not shown). Following over-expression of *eve*, the addition of ChTX is unable to further reduce the magnitude of peak I_Kfast _(62.3 ± 8.4 versus 65.4 ± 4.8 pA/pF; Figure 1c,d). The inability of ChTX to affect I_Kfast _under these conditions is consistent with the reduction of this conductance, due to over-expression of *eve*, being due to a decrease in the current carried primarily by Slo and not by Shal.

### Increased Eve alters action potential firing in motoneurons

**Figure 2:**
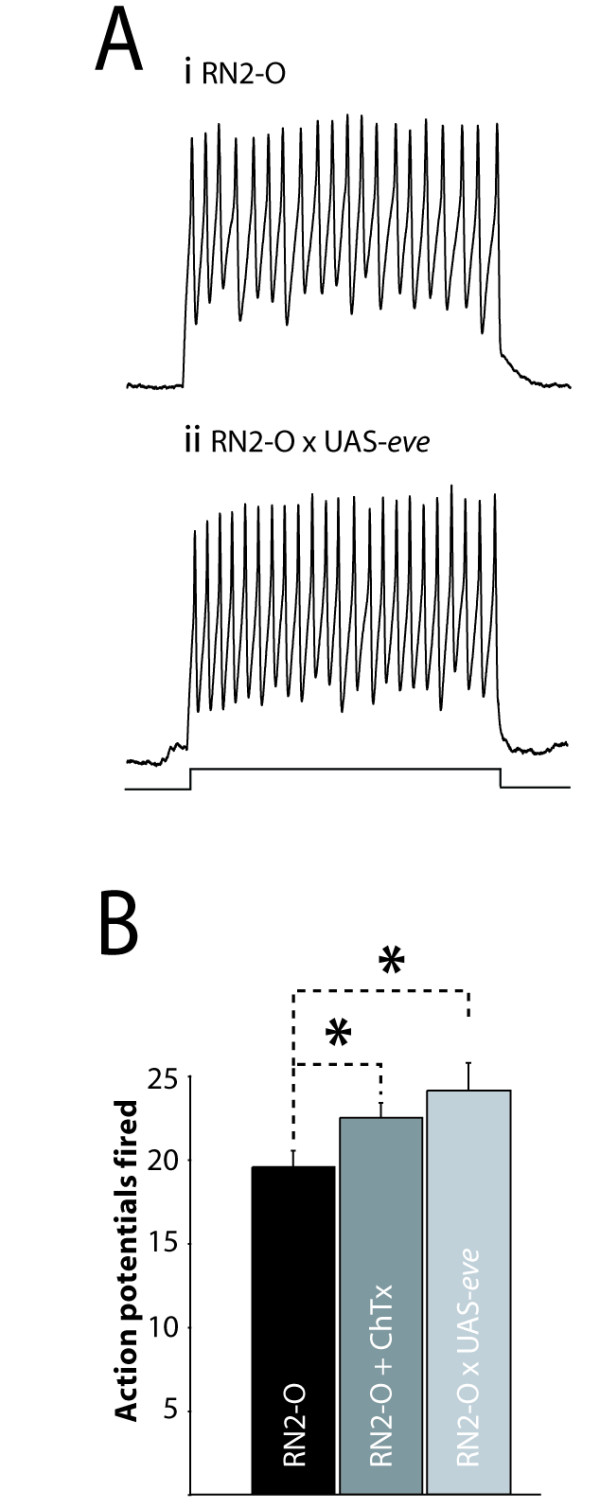
Over-expression of eve increases membrane excitability. **(A) **APs can be evoked in aCC/RP2 by injection of constant current (10 pA/500 ms, indicated by bar). The number of APs fired by aCC/RP2, from a maintained membrane potential of -60 mV, is significantly greater following over-expression of *eve*. **(B) **The average number of APs fired due to injection of constant current (10 pA/500 ms) in control (RN2-O GAL4/+), over-expression of eve or addition of ChTx (200 nM). All values are mean ± SE, n ≥ 8, *p ≤ 0.05.

To test how over-expression of *eve *might impact on functional output, we determined intrinsic membrane excitability (that is, ability to fire action potentials (APs)) by injection of constant current. Our prediction, based on the observed and specific change in I_Kfast _following over-expression of *eve *in aCC/RP2, was that these motoneurons would be more excitable. The membrane potential of individual aCC/RP2 neurons was first maintained at -60 mV by injection of hyperpolarizing current (normal resting membrane potential is -43.5 ± 1.9 mV [22]) and we then used injections of constant depolarizing current (10 pA/500 ms) to induce AP firing. Excitability was determined by counting APs fired. This measure shows that the change in I_Kfast _is indeed associated with a small but significant increase in excitability in aCC/RP2 (19.6 ± 1.0 versus 24 ± 1.6 APs, 10 pA/500 ms, p ≤ 0.05; Figure 2). The membrane potentials induced by this current injection (measured in the presence of 1 mM tetrodotoxin (TTX) to eliminate AP firing) were not significantly different between the two conditions (-33 ± 4 versus -36 ± 2.5 mV, control versus experimental, p > 0.05, data not shown). Thus, there is no obvious change in input resistance that might account for the increased excitability due to depolarizing current injections. Addition of ChTX (200 nM), a specific blocker of I_kslo_, to wild-type aCC/RP2 is also sufficient to induce a comparable increase in membrane excitability (19.6 ± 1.0 versus 22.5 ± 0.9 APs, 10 pA/500 ms, p ≤ 0.05; Figure 1d).

### Increased Eve regulates postsynaptic sensitivity to excitatory neurotransmitter

**Figure 3:**
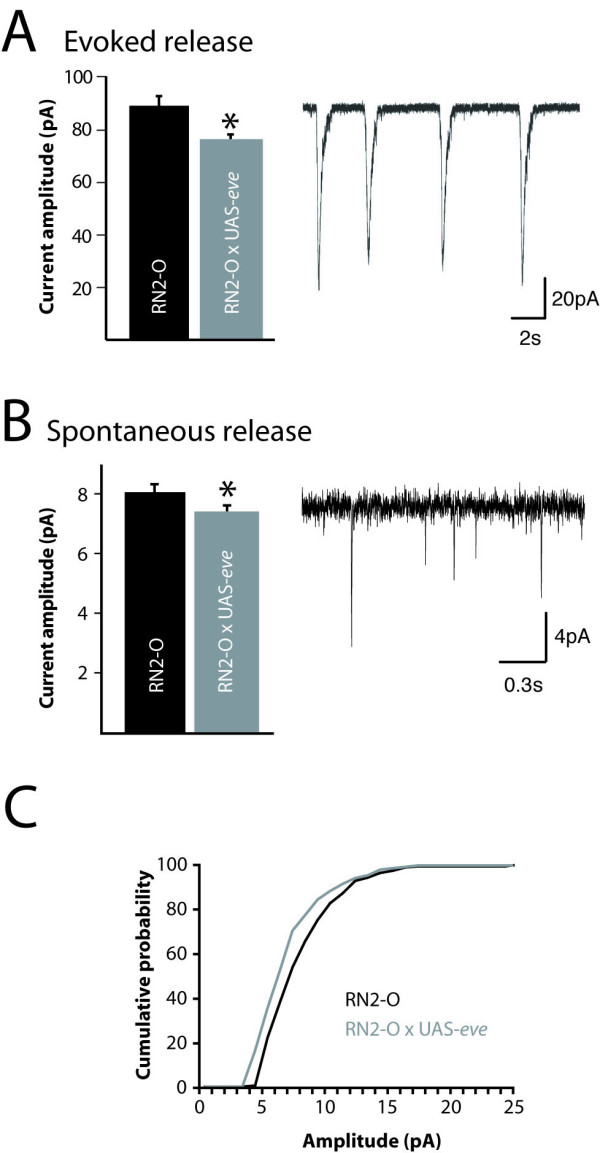
Over-expression of eve reduces sensitivity to ACh. **(A) **Over-expression of *eve *in aCC/RP2 is sufficient to decrease the amplitude of AP-dependent synaptic currents in these neurons (89.2 ± 4.1 versus 76.0 ± 2.5, pA, *p ≤ 0.05). Synaptic currents were recorded, in voltage clamp, from a holding potential of -60 mV. The inset shows a typical recording of synaptic currents from a wild-type aCC neuron (note no differences were observed in current amplitude between aCC and RP2). **(B) **Over-expression of *eve *in aCC/RP2 also results in a significant decrease in the amplitude of AP-independent quantal synaptic currents (8.32 ± 0.26 versus 7.53 ± 0.22 pA, n = 10 cells (200 currents) and 7 (168 currents), respectively, *p ≤ 0.05). The inset shows a typical recording of minis in a control RP2 neuron (note no differences were observed in mini current amplitude between aCC and RP2). All values are mean ± SE (n ≥ 8). **(C) **Cumulative probability plot of amplitudes of AP-independent quantal synaptic currents shows that the majority are smaller in amplitude following over-expression of *eve *compared to control (RN2-O GAL4/+).

We also measured synaptic input to aCC/RP2 following over-expression of *eve *in just these neurons and observed a significant decrease in the amplitude of action potential-dependent synaptic currents (89.2 ± 4.1 versus 76.0 ± 2.5, pA, p ≤ 0.05; Figure 3a). The most parsimonious explanation for this effect, given that expression of *eve *is entirely postsynaptic, is that of reduced postsynaptic sensitivity to ACh. Motoneurons of *Drosophila *first instar larvae receive entirely cholinergic synaptic excitation [23]. To test this prediction, we measured spontaneous miniature (mini) synaptic currents, which are the result of action potential-independent release from presynaptic cholinergic interneurons (that is, the release that persists in the presence of 1 μM TTX). Analysis of minis shows a significant decrease in amplitude in aCC/RP2 in which *eve *is over-expressed compared to controls (8.32 ± 0.26 versus 7.53 ± 0.22, pA, p ≤ 0.05; Figure 3b). Cumulative probability plots, which better show the distribution of mini amplitudes, showed a marked leftward shift, indicating that the large majority of minis recorded exhibit reduced amplitude following over-expression of *eve *(Figure 3c). This reduction is predictive of a decrease in postsynaptic sensitivity for ACh, an effect that is consistent with a reduction of functional cholinergic receptors.

### Levels of Eve are important for setting electrical properties

Given that over-expressing *eve *is sufficient to decrease both I_Kfast _and mini amplitude in aCC/RP2, we wondered whether reducing *eve *expression would result in opposite effects to these two electrical characteristics. Complete removal of *eve *from aCC/RP2, although desirable, is not possible without significantly altering cell morphology, and possibly as an indirect consequence, electrical properties [18] (also ECG Pym and RA Baines, unpublished observations). To circumvent this, we used a temperature-sensitive *eve *allele (*eve*^1*D*19^) previously used by to remove Eve function [15]. We allowed *eve*^1*D*19^/Cyo embryos, maintained at 20°C (permissive temperature) to hatch to first instar larvae (by which time aCC/RP2 have developed their morphology) and then moved some to 30°C (restrictive temperature), while the remainder were kept at 20°C (see Materials and methods for details). We were unable to use homozygous *eve*^1*D*19 ^larvae as these fail to hatch at any temperature (RA Baines, personal observation).

**Figure 4:**
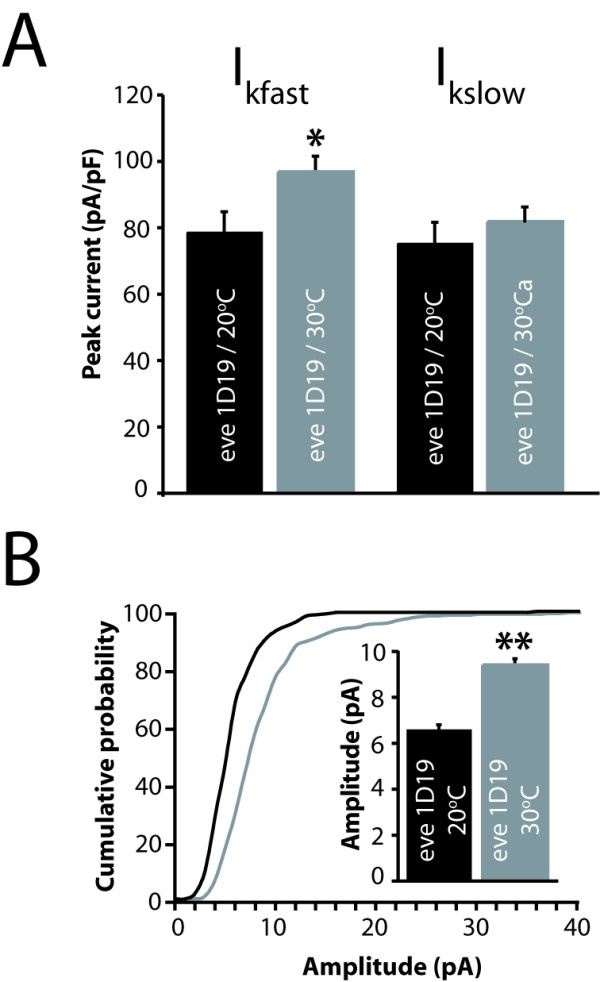
Removal of Eve increases I_Kfast _and sensitivity to ACh. **(A) **Supression of Eve function, using the temperature-sensitive allele *eve*^1*D*19^, results in an increase in I_Kfast _but no change in I_Kslow _(78.5 ± 6.1 versus 96.8 ± 4.6 pA, p < 0.05; see Materials and methods for details). **(B) **Removal of Eve function, through development at 30°C, is also sufficient to increase the amplitude of AP-independent quantal synaptic currents. Cumulative probability plots show that mini currents recorded in aCC/RP2 at 30°C (restrictive temperature, gray line) are greater in amplitude than at 20°C (wild-type Eve level, black line). Inset shows averaged mini current amplitudes (6.6 ± 0.19 versus 9.4 ± 0.26 pA, p < 0.001). Note that the current amplitude in controls (20°C) is smaller in second instars than in first instars (compare Figure 3b), even though the aCC/RP2 neurons are greater in size. This is undoubtedly because the dendritic regions, where these synaptic inputs occur, are further away from the cell body in second instars and, as such, the increased axonal resistance results in smaller quantal currents. All values are mean ± SE, n ≥ 8 cells.

Comparison of both I_Kfast _and mini amplitude in young second instars (approximately 18 h after hatching at 30°C or 30 h at 20°C) shows that both were increased in larvae that developed at 30°C (partial Eve knock-down) relative to those maintained at 20°C (wild-type Eve; Figure 4). By comparison, I_Kslow_, which was not affected by *eve *over-expression (see above), was unaffected by Eve knock-down. That I_Kslow _remains unchanged, together with the fact that cell capacitance was also unchanged (data not shown), serves to demonstrate that development of aCC/RP2 was not disproportionately influenced by developmental temperature. Examination of electrical properties in wild-type larvae similarly showed no temperature-dependent effects (RA Baines, unpublished data). Thus, we conclude that, whilst over-expression of *eve *is clearly sufficient to reduce I_Kfast _and quantal cholinergic currents, a partial removal of Eve results in the opposite phenotype, indicating that the level of Eve function is a critical determinant for the development of these two electrical properties in aCC/RP2.

### Identification of binding sites for Eve

**Figure 5:**
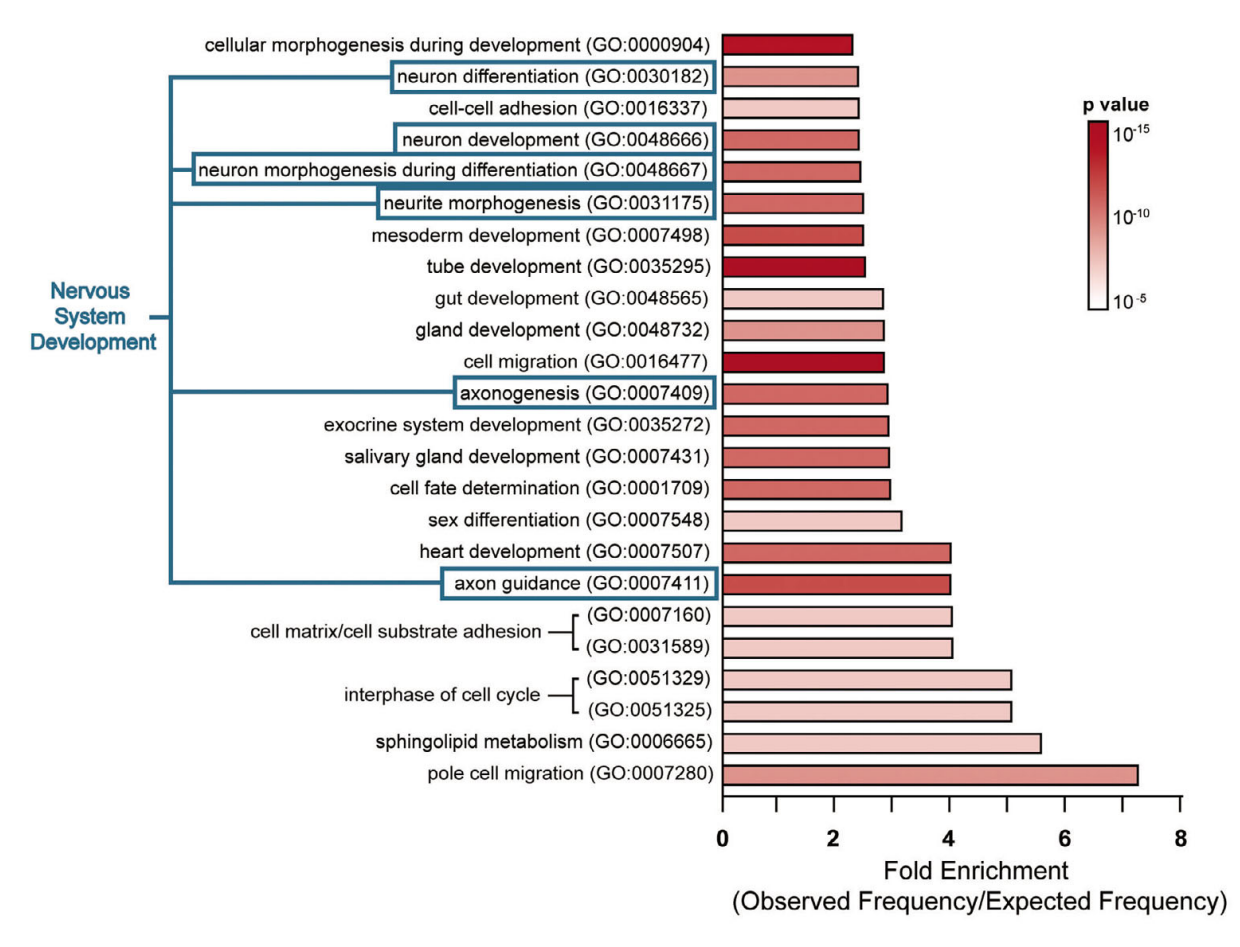
Overview of identified Eve target genes. Unbiased GO annotation overrepresentation analysis of genes near Eve binding sites was performed using GOToolbox [28]. Shown are GO annotation classes that are overrepresented, relative to the annotated genes in the genome, in the list of 819 annotated Eve target genes. Bars represent fold enrichment of the frequency of a class of genes in the list of Eve targets compared to that expected at random. The color of the bar represents the Bonferroni-corrected p-value, indicating the significance of the overrepresentation. A minimum of a 2.5-fold enrichment with a significance level of p < 1 × 10^-5 ^yields a total of 24 overrepresented classes of genes. Eve was previously thought to be involved in nervous system development (blue) and heart development, thereby validating the DamID methodology. The unexpected class of overrepresented genes, such as sphingolipid metabolism, may identify previously uncharacterized roles for Eve.

To identify target genes of Eve, we utilized a technique termed DamID. DamID is an established method for determining the binding sites of DNA- or chromatin-associated proteins [24-26]. Target sites identified by DamID have been shown to match targets identified by ChIP-chip [26] or mapping to polytene chromosomes [27]. We tethered an *Escherichia coli *adenine methyltransferase to Eve. Expression of this fusion protein results in local methylation of DNA that is limited to a few kilobases surrounding its binding sites. These sites are subsequently revealed by cutting DNA with methylation-specific restriction enzymes and hybridizing to microarrays [24,25]. Using tiling arrays that span the entire euchromatic genome (TD Southall, S Choksi, E de Wit, B van Steensel and AH Brand, unpublished data), our analysis identified 2,411 individual DamID peaks in the genome and 1,268 genes (possessing one or more peaks within 2 kb of their transcriptional unit) as potential direct targets of Eve. Using a web-based set of tools, GOToolbox [28], we performed statistical tests to determine Gene Ontology (GO) annotation [29] enrichment on 819 members of our list with associated annotations. Using 'biological process' (GO:0008150) as the broadest classification, we generated a list of overrepresented classes of genes. Figure 5 shows the top overrepresented groups, which include axon guidance (GO:0007411), neuron development (GO:0048666) and heart development (GO:0007507), all of which would be expected from the known function and expression pattern of Eve.

**Figure 6:**
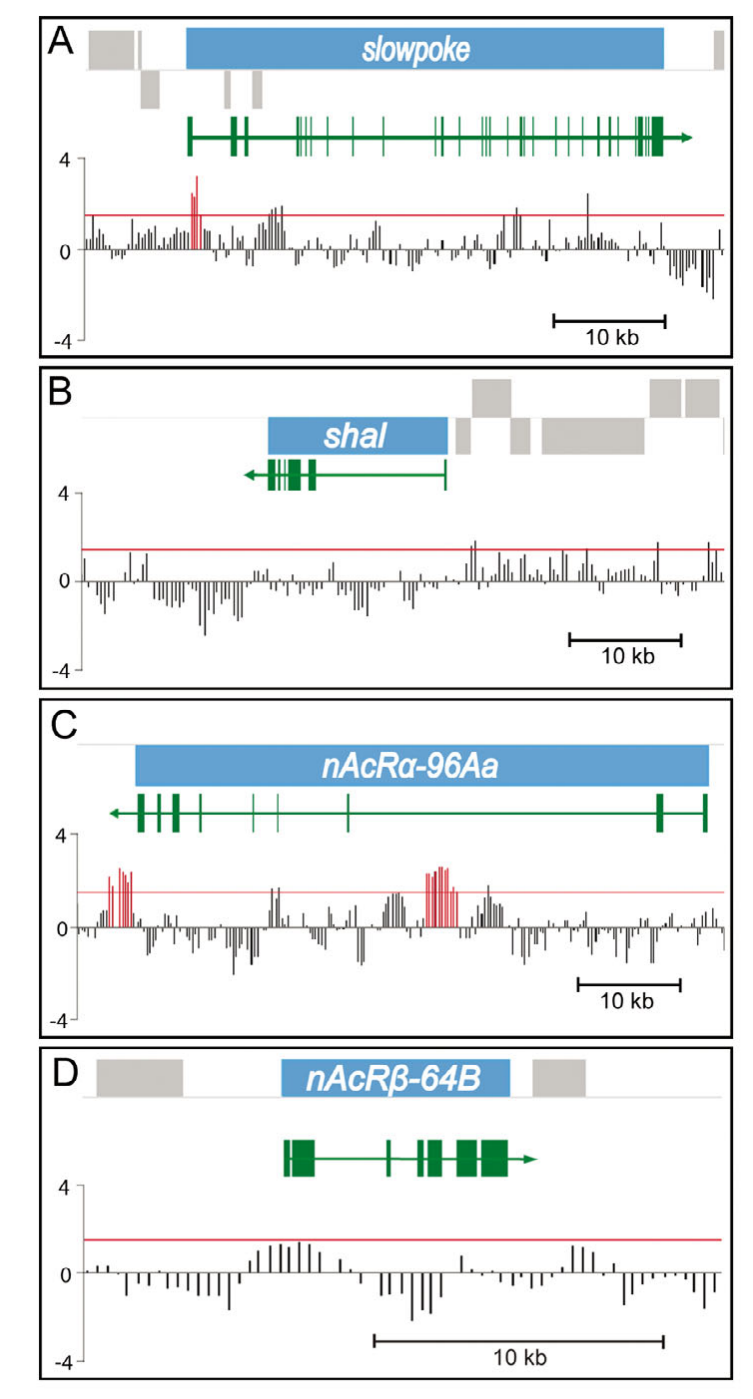
DamID demonstrates direct binding of Eve to *slo *and *nAcRα-96Aa*. The transcription unit of the gene of interest is shown in blue, and other transcription units in the region are shown in grey. Genes depicted above the line are encoded on the forward strand and those below are on the reverse strand. Exons are drawn in green. Vertical bars indicate the position of oligonucleotides on the genomic microarray. Bar heights are proportional to the average of normalized log-transformed ratio of intensities from two replicate DamID *in vivo *binding site mapping experiments. Genomic regions with a Dam-Eve/Dam ratio over 1.4 (red horizontal line) for at least four consecutive probes and with a deconvolved peak height greater than 1.8 were identified as Eve binding sites (red vertical bars).

Our electrophysiological analysis of Eve function implicates *slo *and one or more genes encoding ACh receptors as targets of Eve. Satisfyingly, our analysis identified both *slo *and the nicotinic acetylcholine receptor (nAChR) subunit *nAcRα-96Aa *as containing Eve-binding sites within 2 kb, which, as such, can be considered as putative direct targets of Eve (Figure 6). By contrast, no putative binding sites were identified within 2 kb of *shal*, the product of which also contributes to I_Kfast_. Other functionally relevant ion channel genes identified include *shaker *(does not contribute to I_Kfast _in aCC/RP2; RA Baines, unpublished data) and *shab *(contributes to I_Kslow _at the neuromuscular junction, but unknown contribution within the central nervous system (CNS) [30]).

To demonstrate specificity in gene identification, we analyzed the binding sites for two other, unrelated, transcription factors, Prospero and Asense. Analysis of Prospero binding shows that it is able to bind within 2 kb of *slo *but, importantly, at a different location to that found for Eve. No such binding sites were found for Prospero in *nAcRα-96Aa*. By contrast, analysis of Asense shows no binding within 2 kb of either gene (data not shown). Thus, taken together with these additional controls, we conclude that Eve-DamID identifies both *slo *and *nAcRα-96Aa *as specific putative downstream target genes; an identification that is entirely consistent with our electrophysiology.

### Eve regulates expression of *slo *and *nAcRα-96Aa*

**Figure 7:**
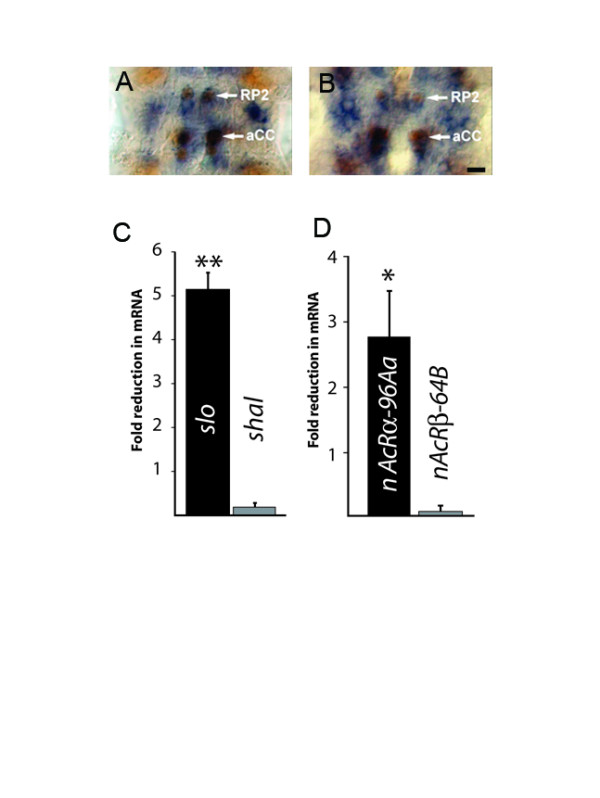
Over-expression of *eve *is sufficient to down regulate expression of *slowpoke *and *nAcRα-96Aa*. **(A,B) ***In situ *analysis shows that aCC/RP2 express both *slo *(a) and *nAcRα-96Aa *(b). A portion of the ventral nerve cord from embryonic stage 15 is shown. Scale bar = 5 μm. **(C) **Pan-neuronal expression of UAS-*eve *is sufficient to significantly decrease the level of *slowpoke *(*slo*) mRNA in isolated CNS from late stage 17 embryos (5.15 ± 0.4-fold decrease, n = 5, **p ≤ 0.01). By comparison, this same manipulation resulted in no significant change in *shal *mRNA (0.2 ± 0.1-fold decrease, n = 5, p > 0.05). **(D) **Pan-neuronal expression of UAS-*eve *results in a significant decrease in the abundance of *nAcRα-96Aa *mRNA (2.78 ± 0.7-fold decrease, n = 5, *p ≤ 0.05), but no change in mRNA for *nAcRβ-64B *(0.1 ± 0.3-fold decrease).

Our identification of both *slo *and *nAcRα-96Aa *as targets of Eve requires that both of these genes are expressed in aCC/RP2. To show this directly, we used *in situ *RNA probes to label transcripts of both genes (Figure 7a,b). Staining for both genes was first evident by about embryonic stage 14 (data not shown) and by stage 15 staining for both *slo *(Figure 7a) and *nAcRα-96Aa *(Figure 7b) was clearly present in the aCC and RP2 motoneurons.

To verify that *slo *and *nAcRα-96Aa *are regulated by Eve, we over-expressed *eve *pan-neuronally (using 1407 GAL4) and used QRT-PCR, which is a more quantitative method than using RNA *in situ *probes, to determine levels of mRNA for each target gene. All values were normalized to *RP49 *mRNA levels to account for differences in starting material. Our prediction, based on the known activity of Eve as primarily a repressor of translation [18], was a reduction in expression of both transcripts. Measurement of *slo *mRNA from isolated late stage 17 embryonic CNS showed a significant reduction following over-expression of *eve *compared to identical measurements made from controls (1407 GAL4/+) (5.15 ± 0.4-fold decrease, p ≤ 0.01). By comparison, no significant change in *shal *mRNA was observed (Figure 7c). Over-expression of *eve *is also sufficient to significantly reduce mRNA abundance for the nAChR subunit *nAcRα-96Aa *(2.78 ± 0.7-fold decrease, p ≤ 0.05). Expression of the *nAcRβ-64B *AChR subunit, the protein product of which is not found together with *nAcRα-96Aa *in functional cholinergic receptors [31], was not affected (Figure 7d). Thus, QRT-PCR confirms that both *slo *and *nAcRα-96Aa *are regulated by Eve, which, taken together with both the presence of Eve-binding sites in these genes and the effect of over-expression on electrical properties, indicates that they are direct targets of this transcription factor.

### The RP motoneurons exhibit different electrical properties to aCC/RP2

**Figure 8:**
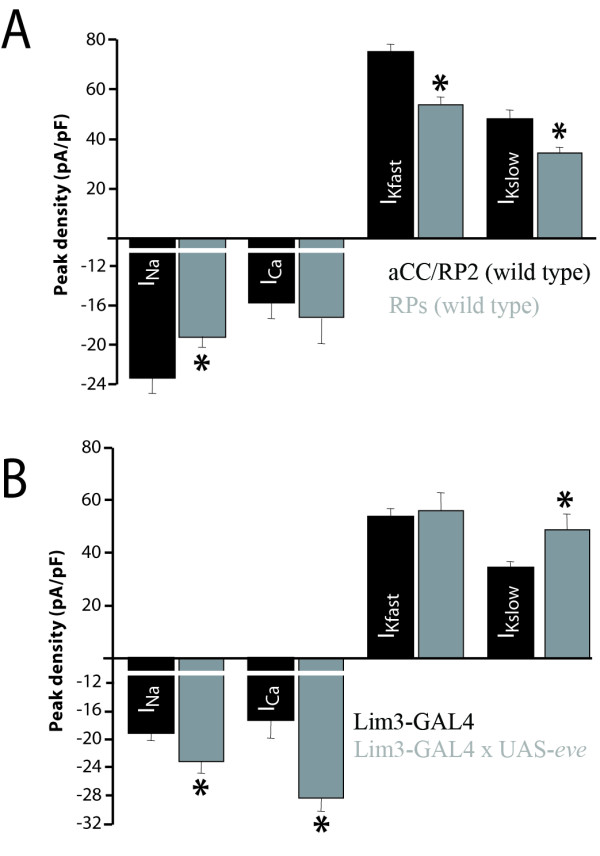
Over-expression of *eve *in the RPs results in altered electrical properties that differ from the effects seen in aCC/RP2. **(A) **In comparison to aCC/RP2, the RP motoneurons (RP1, 3, 4 and 5) exhibit decreased peak conductance in I_Na _(-19.16 ± 1.09 versus -23.36 ± 1.59 pA/pF, *p ≤ 0.05), I_Kfast _(54.1 ± 2.78 versus 78.57 ± 3.96 pA/pF, *p ≤ 0.01) and I_Kslow _(34.7 ± 2.05 versus 48.3 ± 3.5 pA/pF, *p ≤ 0.05), but show no difference in I_Ca _(-17.35 ± 2.53 versus -15.72 ± 1.59 pA/pF, p > 0.05). All values are mean ± SE, with I_Na _n = 11, I_Ca _n = 7, I_Kfast _n = 10, I_Kslow _n = 7. **(B) **Ectopic expression of *eve *in the RPs is sufficient to increase I_Na _(-19.2 ± 1.09 versus -23.2 ± 1.60 pA/pF, *p ≤ 0.05), I_Ca _(-17.35 ± 2.53 versus -28.4 ± 1.81 pA/pF, **p ≤ 0.01) and I_Kslow _(34.7 ± 2.05 versus 49.2 ± 5.75 pA/pF, *p ≤ 0.05) but did not change I_kfast _(54.1 ± 2.78 versus 57.6 ± 3.95 pA/pF). **(C) **Expression of *eve *in the RPs results in an increase in membrane excitability (18.5 ± 0.98 versus 23.75 ± 0.99, action potentials fired, 10 pA/500 ms, **p ≤ 0.01) and **(D) **a decrease in amplitude of action potential-dependent synaptic currents (93.25 ± 2.0 versus 74.08 ± 0.06 pA, ***p ≤ 0.001). All values are mean ± SE, with I_Na _n = 9, I_Ca _n = 7, I_Kfast _n = 10, I_Kslow _n = 7, membrane excitability n = 8, synaptic input n = 10 cells.

Previous work has shown that the aCC/RP2 motoneurons, which are both *eve*-positive, exhibit near identical electrical properties [4]. We reasoned that other motoneurons that do not express *eve *might exhibit different electrical properties from aCC/RP2. To test this we characterized the *isl*, *Hb9 *and *lim3 *positive motoneurons RP1, 3, 4 and 5 (hereafter termed the RPs), identified using *lim3*-GAL4 to drive UAS-GFP(*nls*) [32]. Interestingly, all of the RPs exhibited near identical electrical properties; thus, although we were able to identify which RP neuron was recorded from by dye-labeling, we were unable to separate out each individual RP neuron type based on analysis of electrical properties. Though indistinguishable from one another, electrical characteristics of the RPs markedly differed from those of aCC/RP2 (Figure 8a). Specifically, the RPs exhibit significantly reduced peak I_Na_, I_Kfast _and I_kslow_, but no change in I_Ca _compared to aCC/RP2. By comparison, membrane excitability and amplitude of action potential-dependent synaptic currents were not significantly different (data not shown). Thus, two groups of motoneurons (aCC/RP2 and RPs) that differ in their morphology and transcription factor expression also differ in their electrical properties. However, although the underlying voltage-gated conductances exhibit significant differences, the action potential output of these motoneurons would seem to be similar, indicative perhaps of additional regulatory mechanisms.

### Over-expression of *eve *in RP motoneurons results in altered electrical properties

An important question raised by our findings is how universal the effects of over-expressing *eve *are? Specifically, can *eve *expression affect other motoneurons in the same manner as aCC/RP2 or is its ability to influence electrical properties cell-type specific? To test this, we over-expressed *eve *in the RPs (using *lim3*-GAL4) that do not normally express this transcriptional repressor. Our results clearly show that over-expression of *eve *in the RPs does indeed alter their electrical properties, but that the changes observed do not recapitulate the changes seen following over-expression in aCC/RP2. Thus, expression of *eve *in the RPs is sufficient to produce significant increases in I_Na_, I_Ca _and I_Kslow _(Figure 8b). Perhaps more notably, I_Kfast_, which is reduced by over-expression of *eve *in aCC/RP2, remains unaffected by ectopic expression of *eve *in the RPs. By contrast, expression of *eve *in the RPs, similar to that observed in aCC/RP2, is sufficient to increase membrane excitability (18.5 ± 0.98 versus 23.75 ± 1.0 APs, 10 pA/500 ms, p ≤ 0.05, data not shown) and to reduce the amplitude of action potential-dependent synaptic currents (93.24 ± 2.0 versus 74.08 ± 1.42 pA, p ≤ 0.001, data not shown). Given that changes to voltage-gated currents observed in the RPs differ from those in aCC/RP2, we conclude that the ability of *eve *to regulate electrical properties is cell-type specific.

### Over-expression of *islet *or *Hb9 *in aCC/RP2 results in altered electrical properties

**Figure 9:**
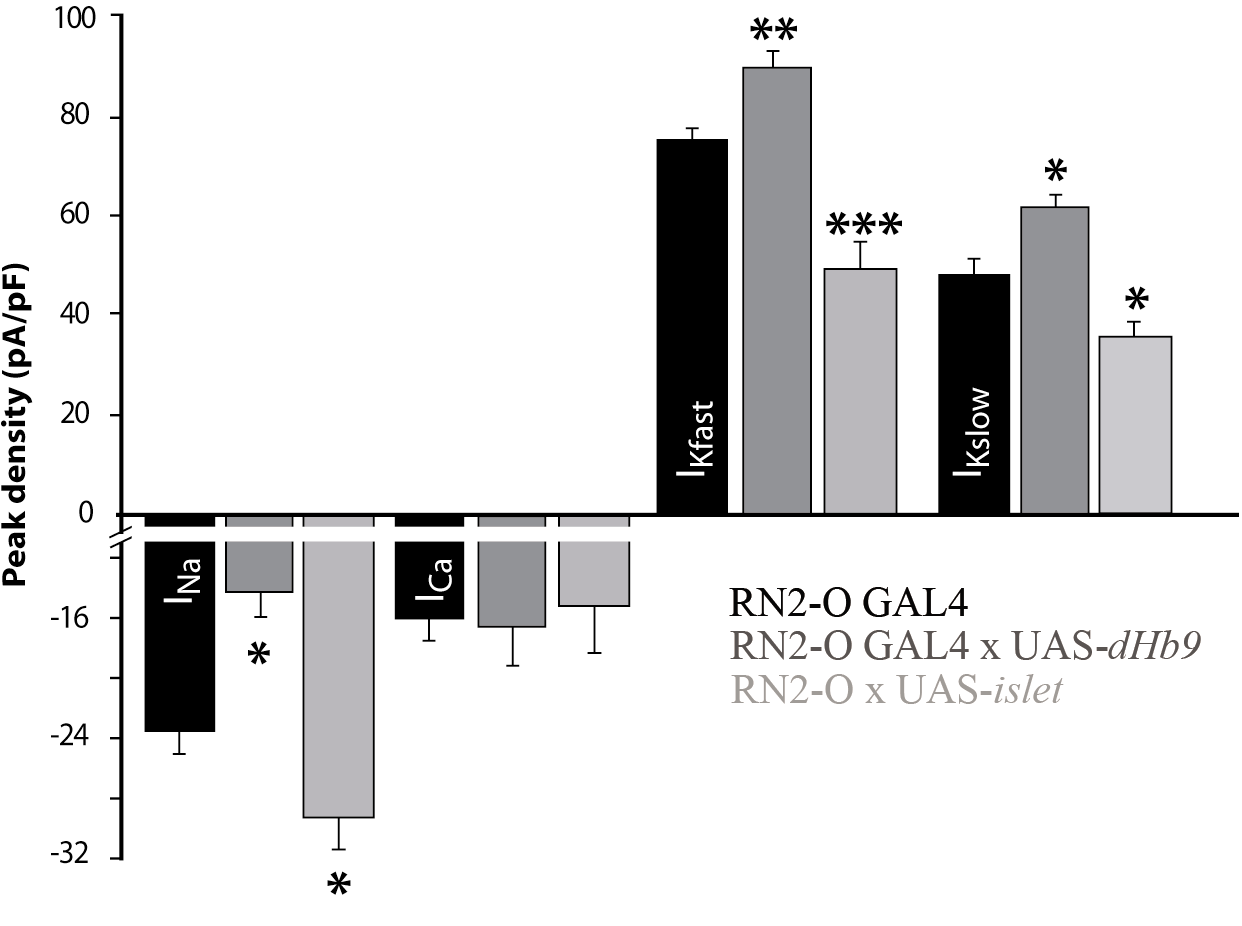
Increased expression of *isl *or *dHb9 *in aCC/RP2 results in cell-specific perturbations in electrical properties. **(A) **Over-expression of *isl *in aCC/RP2 is sufficient to increase I_Na_, reduce both I_Kfast _and I_kslow _while having no effect on I_Ca _(-23.4 ± 1.6 versus -29.4 ± 2.2 pA/pF; -15.7 ± 1.6 versus -15.0 ± 3.23 pA/pF; 75.2 ± 3.0 versus 49.4 ± 5.7 pA/pF; 49.15 ± 5.75 versus 35.83 ± 3.13 pA/pF for I_Na_, I_Ca_, I_Kfast _and I_kslow_, respectively). **(B) **Ectopic expression of *dHb9 *in aCC/RP2, by contrast, results in a decrease in I_Na_, no change in I_Ca _and significant increases in both I_Kfast _and I_Kslow _(-23.4 ± 1.6 versus -14.0 ± 1.75 pA/pF; -15.7 ± 1.6 versus -16.4 ± 2.61 pA/pF; 54.1 ± 2.78 versus 57.51 ± 3.8 pA/pF; 34.7 ± 2.05 versus 42.15 ± 4.89 pA/pF for I_Na_, I_Ca_, I_Kfast _and I_kslow_, respectively). All values are mean ± SE, with *islet *(I_Na _n = 7, I_Ca _n = 7, I_Kfast _n = 9, I_Kslow _n = 7) and *Hb9 *(I_Na _n = 9, I_Ca _n = 6, I_Kfast _n = 12, I_Kslow _n = 7). *P ≤ 0.05, **p ≤ 0.01.

Our results are consistent with a link between the determination of morphology and determination of electrical properties through the transcription factor Eve. To test if other transcription factors demonstrated to influence neuronal morphology also regulate electrical properties we ectopically expressed *isl *or *Hb9 *in aCC/RP2 (using RN2-O GAL4), and characterized the consequences on electrical properties. It should be noted that, although over-expression of either *isl *or *Hb9*, similar to *eve*, has previously been shown to alter axonal trajectory in motoneurons at earlier stages [14,16], we saw no obvious alterations in central dendritic morphology under these conditions (data not shown). Expression of either *isl *or *Hb9 *in aCC/RP2 resulted in significant alterations to normal electrical properties (Figure 9). Ectopic expression of *isl *is sufficient to increase I_Na _and decrease I_Kfast _and I_Kslow _with no change to I_Ca _(Figure 9). By comparison, ectopic expression of *Hb9 *resulted in a significant decrease in I_Na _and increases in both I_Kfast _and I_Kslow _but again no change to I_Ca _(Figure 9).

The cumulative effects of specific changes to individual current densities on membrane excitability following mis-expression of *isl *or *Hb9 *are more consistent. Neither transcription factor affected intrinsic membrane excitability in aCC/RP2 (data not shown). The general conclusion that we draw from this data set is that the effect of over-expression of *isl *or *Hb9 *(and indeed *eve*) is cell-type specific, which is entirely predictive for a combinatorial code of regulation of neuronal electrical properties.

## Discussion

The emergence of appropriate behavior depends on the maturation of neuronal circuits. For this to occur the constituent neurons must not only develop a specific pattern of dendritic branching and synaptic connectivity, but must also express a stereotyped mix of ion channel genes that, together, set membrane excitability. Previous work in *Drosophila *has shown that motoneuron axon pathfinding is determined by a combinatorial code of transcription factor expression, including Eve, Islet and Hb9 [14-16,18,33]. In the present study, we show that at least one of these transcription factors, pivotal for morphological development, is also sufficient to affect the acquisition of electrical properties. It is well established that cellular morphology and electrical properties are key contributors to the diversity in neuronal signaling observed in the CNS. Against this backdrop it is gratifying that specific transcription factors are clearly able to regulate both these facets, which suggests that each are regulated by common developmental mechanisms.

Our electrophysiology clearly demonstrates that over-expression of *eve *in aCC/RP2 results in a down-regulation of both peak I_Kfast _and amplitude of cholinergic minis. I_Kfast _is a composite of at least two separate conductances encoded by *shal *and *slo *[19,20,34]. Addition of ChTX indicates that the effect of Eve on I_Kfast _is through a reduction in the Slo-mediated component of this current. This conclusion is reinforced by two complementary approaches: DamID and QRT-PCR, the latter of which shows that *slo *is specifically repressed by Eve while *shal *remains unchanged. That both DamID and QRT-PCR identify *slo *as a target of Eve repression indicates that regulation of this ion channel gene is direct (that is, not occurring indirectly through unknown intermediates). Transcriptional regulation of *slo *is relatively complex [35], with at least five different promoters characterized: C_0 _and C_1_, which drive expression in neurons; C_1b _and C_1c_, which drive expression in the mid-gut; and C_2_, which drives expression in muscle and trachea. Each promoter gives rise to different splice variants. It remains to be determined how Eve regulates the expression of individual splice variants. The family of channels of which *slo *is a member, BK Ca^2+^-gated potassium channels, are activated by membrane depolarization during action potential firing and, as such, contribute to the after-hyperpolarization [36]. Consistent with our data, I_kslo _has been shown to regulate excitability via both duration and frequency of action potential firing [37] and also to modulate synaptic release [38].

In addition to decreasing peak I_Kfast_, over-expression of *eve *in aCC/RP2 is sufficient to decrease the amplitude of action potential-dependent excitatory synaptic currents. Analysis of spontaneous minis reveals a similar reduction, which is indicative of reduced postsynaptic sensitivity to ACh. Our analyses indicate that expression of *nAcRα-96Aa*, a nAChR subunit, is negatively regulated by Eve. Unlike *slo*, however, we have not been able to use additional pharmacology to further corroborate this regulation. To show specificity, however, we used QRT-PCR to analyze mRNA levels for a second nAChR, *nAcRβ-64B*, a subunit not found in the same complex as nAcRα-96Aa [39]. We saw no significant alterations in *nAcRβ-64B *mRNA levels, which allows us to tentatively conclude that Eve acts selectively on *nAcRα-96Aa*. This regulation, which is evident from DamID, QRT-PCR and electrophysiology, is again likely to be direct.

There is, however, a caveat that should be borne in mind in the interpretation of our data. This is that the changes we observed following over-expression of *eve *may be the average of both direct and indirect effects. We consider that indirect effects are likely to arise through two main sources. The first possibility would be changes that result from homeostatic compensatory mechanisms that are known to be active in *Drosophila *motoneurons [4,22]. Such homeostatic mechanisms are capable of adjusting the relative peak amplitudes of specific membrane conductances (most notable I_Na _and I_K_) to maintain consistency in action potential firing [4,40]. Thus, changes to membrane excitability mediated by *eve *over-expression may be countered, at least in part, by homeostatic regulation. The effects observed in action potential firing following over-expression of *eve *in aCC/RP2 are in keeping with the known homeostatic mechanisms active in these two motoneurons; a decrease in exposure to synaptic excitation results in a compensatory increase in excitability to fire action potentials [4]. The changes to the underlying electrical properties that are associated with this response (that is, a decrease in I_Kfast_) are not, by comparison, in keeping with known homeostatic regulation that would predict significant increases in I_Na_, I_Kfast _and I_Kslow_. Thus, whilst homeostatic regulation may contribute to the changes observed in membrane excitability, it seems unlikely that such mechanisms can account entirely for the changes observed in specific underlying membrane conductances.

A second possibility that may result in indirect change to membrane excitability is an alteration in the synaptic connectivity of motoneurons in which *eve *is over-expressed. Considering the documented role for *eve *in axon pathfinding, it is conceivable that alterations in electrical properties may arise as secondary consequences of morphological changes. We tend to discount this possibility for two reasons. First, all motoneurons were filled during our recordings and no obvious perturbation in wild-type morphology was noted. Of course, our level of analysis would not have detected subtle changes in fine dendritic branching. Second, Landgraf *et al*. [15] show that misexpression of *eve *is sufficient to only perturb axon pathfinding but not final target recognition in motoneurons (which is only delayed). Thus, although over-expression of *eve *in the ventral motoneurons is sufficient to misdirect their axons to dorsal muscles, these errors are rectified by the time of hatching.

In addition to our own experiments, there is a precedent for transcription factor misexpression resulting in altered membrane excitability. For example, expression of *lox1*, a homologue of the *Drosophila *genes *sex-combs reduced *and *antennapedia*, in leech neurons is sufficient to increase the size of the AP [41]. In keeping with a basic principle of a combinatorial code, these authors report cell-type specific effects – only discrete groups of cells exhibit increased action potential amplitude. In the ascidian, *Halocynthia roretizi*, motoneurons that express the transcription factor HRlim have a set of common properties, including the expression of the sodium channel gene *TuNa2 *[42]. Ectopic expression of HRlim is sufficient to drive expression of TuNa2 in cells that normally do not express either.

An important question raised by our examination of the consequences of *eve *over-expression in differing motoneuron subpopulations is the universality of its effects. Our data are consistent with a model in which the transcriptome of specific cell types is a critical determinant of the nature of regulation. Comparison of the effects produced by over-expression of *eve *in aCC/RP2 compared to those produced in the RPs shows differences consistent with this hypothesis. Whatever the precise mechanism, this apparent dichotomy of action strongly implicates a context-dependent mode of regulation. Such a conclusion is not too surprising given that Eve, Isl and Hb9 form part of a combinatorial code of motoneuron specification [9] and that specification of other neuronal properties, for example, *dFMRFa *expression and *furnin 1 *expression, is also under complex combinatorial and non-combinatorial control [33]. Thus, the electrical properties of neuronal membranes are most likely dictated by a combined activity of a range of transcription factors, which our data indicates includes Eve, Islet and HB9. Alteration of this balance within specific neurons will likely result in an equally diverse range of effects on both electrical and signaling properties.

## Conclusion

The processes by which neurons acquire their unique electrical characteristics are, at present, not particularly well understood. Our study is of importance because it shows that a transcription factor known to be essential for axon guidance decisions is also able to influence the acquisition of specific electrical properties in the same motoneurons. Based on this observation, it is our prediction that other members of the combinatorial code (Lim3, Islet, and so on) will act through the transcriptional regulation of additional sets of ion channel genes, the particular expression of which will account for the unique electrical characteristics that we observe between motoneurons. It will now be of importance to determine how Eve influences the expression of ion-channel genes when expressed in other, non-endogenously *eve *expressing motoneurons. Our results clearly indicate that the effects we observe in aCC/RP2 are not recapitulated when *eve *is ectopically expressed in the RP1,3–5 motoneurons, indicative perhaps that additional co-factors might be required. Presumably, these co-factors might also be cell-type specific.

## Materials and methods

### Fly stocks

Flies were maintained on apple juice agar supplemented with yeast at 25°C. Wild type (WT) was Canton-S. 1407 GAL4 (homozygous second chromosome) was used to express UAS transgenes in all CNS neurons. RN2-O GAL4 (homozygous viable second chromosome) was used to selectively express UAS transgenes in aCC and RP2 [22,43]. Lim-3 GAL4 was used to drive expression in the RP1,3–5 motoneurons [32]. UAS-*eve *on TM6/P [rosy+{l(3)}], was re-balanced on a TM3 GFP balancer. UAS-*islet *was kindly provided by Stephan Thor [14] and UAS-*dHb9 *was kindly provided by James Skeath [44]. To remove Eve function a temperature-sensitive allele, *eve*^1*D*19^, was used [15]. Mated females were allowed to lay, and embryos to develop, at 20°C (permissive temperature), The resultant first instar larvae (which were all *eve*^1*D*19^/Cyo act::GFP) were either placed at 30°C (restrictive temperature) for approximately 18 h or maintained at 20°C. Recordings were made from aCC/RP2 in young second instar larvae. Homozygous *eve*^1*D*19 ^embryos fail to hatch even at the permissive temperature.

### Electrophysiology

All of the recordings were performed in young first instar larvae, 1 to 4 h after hatching, at room temperature (22 to 24°C). Whole-cell recordings (current and voltage clamp) were achieved using thick-walled borosilicate glass electrodes (GC100TF-10; Harvard Apparatus, Edenbridge, UK), fire polished to resistances of between 15 and 20 MΩ. Cells were initially identified based on position within the ventral nerve cord and absolute identification was determined after recording by labeling with sulfur rhodamine (0.3%; Molecular Probes, Eugene, OR, USA), which was included in the patch saline. Recordings were made using an Axopatch-1D amplifier controlled by pClamp 8.1 (Axon Instruments, Foster City, CA, USA). Only cells with an input resistance >1 GΩ were accepted for analysis. Traces were filtered at 2 kHz and sampled at 20 kHz. To better resolve Na^+ ^currents, an on-line leak subtraction protocol was used (P/4). Currents shown are the averages of five trials for each cell. Membrane excitability was determined using injection of depolarizing current (10 pA/500 ms) from a maintained membrane potential (Vm) of -60 mV. Vms were maintained at -60 mV by injection of a small amount of hyperpolarizing current. Input resistance, which was determined by injection of 0.5 pA hyperpolarizing current, remained statistically unchanged in all of the genetic backgrounds tested (WT = 7.2 ± 0.9 GΩ). To determine the effect of gene expression on electrical properties we analyzed the peak conductance for each current analyzed (I_Na _at -15 mV, I_Ca _at 0 mV, and I_Kfast _and I_Kslow _at +45 mV).

### Solutions

External saline for dissection and current clamp analysis of excitability consisted of the following (in mM): 135 NaCl, 5 KCl, 4 MgCl_2_·6H_2_O, 2 CaCl_2_·2H_2_O, 5 N-Tris [hydroxymethyl]methyl-2-aminoethanesulfonic acid (TES), 36 sucrose. For isolation of K^+ ^currents, the following solution was used (in mM): 135 NaCl, 5 KCl, 4 MgCl_2_·6H_2_O, 2 CaCl_2_·2H_2_O, 5 TES, 36 sucrose and 1 μM TTX (Alomone Labs, Jerusalem, Israel). For isolation of Ca^2+ ^currents, the following solution was used (in mM): 50 NaCl, 6 KCl, 50 tetraethylammonium chloride (TEA), 50, BaCl, 10 4-AP, 10 HEPES, 10 glucose, 10 MgCl_2_·6H_2_O, and 1 μM TTX. For isolation of Na^+ ^currents, the following solution was used (in mM): 100 NaCl, 5 KCl, 50 TEA, 10 4-AP, 10 HEPES, 10 sucrose, and 0.5 CaCl_2_·2H_2_O. All of the solutions were pH 7.15.

Internal patch solution consisted of (in mM): 140 K^+ ^methylsulfonate (KCH_3_SO_3_), 2 MgCl_2_·6H_2_O, 2 EGTA, 5 KCl, and 20 HEPES, pH 7.4. When recording Na^+ ^currents, CsCl_2 _was substituted for KCH_3_SO_3_.

### Plasmid construction for DamID

The pUASTNDam-*eve *construct was made by PCR amplification of *eve *cDNA from a *Drosophila *embryonic cDNA library and insertion into pUASTNDam [45] using *Bgl*II and *Not*I sites. The *eve *cDNA was amplified using the following primers: forward (5'-GGGAGATCTGATGCACGGATACCGAACCTACAAC-3') and reverse (5'-GTACGCGGCCGCTTACGCCTCAGTCTTGTAGGGCTTG-3'). Transgenic flies containing pUAST-NDam-*eve *were generated as described previously [46], except that DNA was prepared using a Qiagen (Crawley, UK) miniprep kit.

### Preparation of Dam-methylated DNA

Stage 17 embryos (16 to 22 hours after egg-laying (AEL)) were collected from UAS-Dam (control) and UAS-Dam-*eve *flies. Genomic DNA was isolated from embryos using the Qiagen DNeasy kit. Embryos (50 mg) were hand homogenized in 180 μl of phosphate-buffered saline, then 4 μl of RNase A (100 mg/ml) was added and left to incubate for two minutes to remove RNA from the sample. DNA digestion and PCR amplification was performed as previously described [45].

### DamID analysis

To map binding sites on a genome-wide scale, we utilized whole genome 375,000 feature tiling arrays, comprising 60-mer oligonucleotides spaced at approximately 300 bp intervals, designed against Release 4.0 of the *Drosophila *genome [45]. The control and experimental samples were labeled and hybridized to these custom arrays. Arrays were then scanned and intensities extracted (Nimblegen Systems, Madison, WI, USA). Two replicates of the Dam-*eve *versus Dam only comparison were performed. Log_2 _ratios of each spot were normalized using a median-centering normalization. Normalized ratios were averaged across both slides. Peaks were identified. A peak-finding algorithm was used to identify regions of the genome bound by Eve, with parameters as follows: peak height threshold ≥1.8 log-fold change, in addition to the surrounding probes (approximately 1,200 bp) possessing a value ≥1.4 log-fold change (PERL scripts for analysis available upon request). GO annotation over-representation analysis was performed using GOToolbox [28]. Parameters for over-representation searches include: Biological Process, specificity = 3, Hypergeometric testing, and Bonferroni-correction of p-values.

### *In situ *hybridization and immunohistochemistry

*In situ *hybridization was performed as previously described [45], using a hybridization temperature of 65°C. The primers used to generate the RNA probes are as follows: slo-for, GAAGGACTTTGATTTCGAGAAGAC; slo-revT7, CAGTAATACGACTCACTATTATCAAGAGTTATCATCCTTGTTGGA; als-for, ACAACATTCAAGACTGCTAACTGG; als-revT7, CAGTAATACGACTCACTATTACTGATGTTGCAGTTGCTGTTG.

Immunohistochemistry was performed after the *in situ *protocol using an Eve antibody at 1:5,000 [17] and developed using 3,3'-diaminobenzidine (DAB).

### RNA extraction and real time PCR

Details of the real time PCR method are described in [47]. PCR primers (forward and reverse primers in 5' to 3' orientation) were as follows: rp49, CCAAGGACTTCATCCGCCACC and GCGGGTGCGCTTGTTCGATCC; eve, CAATCCGCTCCATCAGTTCC and CCGCAATCACAGTTGTCGTC; nAcRβ-64B, TCCTGGCATCCAACGTTTCG and TTTCGGGCAATCGCAAGTCG; nAcRα-96Aa, CAGCGAGAACACCTTATAGC and GTACAACGCCGAGGAAATAC; slo, CGTTTGCGTCCGCAAGGAGCCGGACC and GGAAGTCCTTCAGGAAATGCGACACGG; shal, ATGGCCAACGTGGTGGAGACGGTGCCGTGTGG and TTCGCTGGCGCAGGACTTGAGCGTGTAGCC.

Each reaction contained 5 μl of PCR Master Mix (3 mM MgCl_2_, dNTPs, *Taq *polymerase; Biogene, Cambridge, UK), 500 nM forward and reverse primers, 1 μl of cDNA, and 1:1,000 dilution of SYBR Gold (Biogene) made up to 10 μl with PCR-grade water. PCR was performed using a Roche LightCycler (Roche, Lewes, UK). After an initial denaturation step at 94°C for 60 s, temperature cycling was initiated. Each cycle consisted of denaturation at 94°C for 0 s, hybridization at 54°C for 5 s for *eve*, 57°C for *nACh receptors*, 60°C for *slo *and 65°C for *shal *and elongation at 72°C for 10 s. Determination of *rp49 *was performed at all temperatures. The fluorescent signal was acquired at the end of each hybridization step (F2/F1 channels, fluorimeter gains regulated on 1 for F1, 1 for F2, and 1 for F3). A total of 30 cycles was performed. The authenticity of the PCR products was verified by melting-curve analysis and the presence of a single band of appropriate molecular size by agarose gel electrophoresis. Relative quantification for any given gene, expressed as fold variation over control, was calculated from the determination of the difference between the Ct of the test gene and that of the calibrator gene (*rp49*). Ct values used were the means of triplicate replicates. Experiments were repeated at least five times.

### Statistics

Electrophysiology and QRT-PCR data were compared using a non-paired *t *test. Results were deemed significant at p ≤ 0.05. All of the values shown are mean ± SE.

## Competing interests

The author(s) declare that they have no competing interests.

## Authors' contributions

ECGP performed electrophysiology, analyzed data, and wrote the paper. TDS performed DamID and *in situ *protocols, and analyzed data. CJM performed PCR. AHB designed the research and wrote the paper. RAB performed electrophysiology, designed the research, analyzed data, and wrote the paper. All authors read and approved the final manuscript.
